# Radionuclide generator-based production of therapeutic ^177^Lu from its long-lived isomer ^177m^Lu

**DOI:** 10.1186/s41181-019-0064-5

**Published:** 2019-07-15

**Authors:** Rupali Bhardwaj, Hubert Th. Wolterbeek, Antonia G. Denkova, Pablo Serra-Crespo

**Affiliations:** 1 Applied Radiation and Isotopes, Department of Radiation Science and Technology, Faculty of Applied Sciences, Technical University Delft, Mekelweg 15, 2629 JB, Delft, The Netherlands; 2 Catalysis Engineering, Department of Chemical Engineering, Faculty of Applied Sciences, Delft University of Technology, Van der Maasweg 9, 2629 HZ Delft, The Netherlands

**Keywords:** Lutetium-177, ^177m^Lu/^177^Lu radionuclide generator, Nuclear isomer separation, ^177^Lu production

## Abstract

**Background:**

In this work, a lutetium-177 (^177^Lu) production method based on the separation of nuclear isomers, ^177m^Lu & ^177^Lu, is reported. The ^177m^Lu-^177^Lu separation is performed by combining the use of DOTA & DOTA-labelled peptide (DOTATATE) and liquid-liquid extraction.

**Methods:**

The ^177m^Lu cations were complexed with DOTA & DOTATATE and kept at 77 K for periods of time to allow ^177^Lu production. The freed ^177^Lu ions produced via internal conversion of ^177m^Lu were then extracted in dihexyl ether using 0.01 M di-(2-ethylhexyl) phosphoric acid (DEHPA) at room temperature. The liquid-liquid extractions were performed periodically for a period up to 35 days.

**Results:**

A maximum ^177^Lu/^177m^Lu activity ratio of 3500 ± 500 was achieved with [^177m^Lu]Lu-DOTA complex, in comparison to ^177^Lu/^177m^Lu activity ratios of 1086 ± 40 realized using [^177m^Lu]Lu-DOTATATE complex. The ^177^Lu-^177m^Lu separation was found to be affected by the molar ratio of lutetium and DOTA. A ^177^Lu/^177m^Lu activity ratio up to 3500 ± 500 was achieved with excess DOTA in comparison to ^177^Lu/^177m^Lu activity ratio 1500 ± 600 obtained when lutetium and DOTA were present in molar ratio of 1:1. Further, the ^177^Lu ion extraction efficiency, decreases from 95 ± 4% to 58 ± 2% in the presence of excess DOTA.

**Conclusion:**

The reported method resulted in a ^177^Lu/ ^177m^Lu activity ratio up to 3500 after the separation. This ratio is close to the lower end of ^177^Lu/^177m^Lu activity ratios, attained currently during the direct route ^177^Lu production for clinical applications (i.e. 4000–10,000). This study forms the basis for further extending the liquid-liquid extraction based ^177m^Lu-^177^Lu separation in order to lead to a commercial ^177m^Lu/^177^Lu radionuclide generator.

**Electronic supplementary material:**

The online version of this article (10.1186/s41181-019-0064-5) contains supplementary material, which is available to authorized users.

## Background

Radionuclide generators are known to have brought revolutionary opportunities in the development of nuclear medicine (Knapp & Dash, 2016; Knapp & Mirzadeh, 1994; Knapp et al., 2014; Knapp & Baum, 2012). The current state of the art of ^99m^Tc, ^188^Re, ^68^Ga pharmaceuticals owes their existence largely to the availability of their corresponding radionuclide generators (Roesch & Riss, 2010; Pillai et al., 2012). They offer continuous, on-site and on-demand isolation of a short-lived daughter radionuclide from its longer-lived mother radionuclide. Lutetium-177 (^177^Lu) is a radionuclide that could also benefit from the advantages of a generator vastly. ^177^Lu is well-known for its theranostic potential and is expected to play a crucial role in fulfilling the global demand of radionuclides for many targeted radionuclide therapy applications (Das & Banerjee, 2016; Das & Pillai, 2013). The [^177^Lu]Lu-DOTATATE has already been FDA approved for the application in neuroendocrine tumour therapy (https://www.fda.gov/NewsEvents/Newsroom/PressAnnouncements/ucm594043.htm, n.d.). Currently, other ^177^Lu radiopharmaceuticals have also entered the clinic in the treatment of prostate cancer, lung cancer, non-Hodgkin lymphoma, bone pain palliation and others (Banerjee et al., 2015; Emmett et al., 2017; Hofman et al., 2018; Repetto-Llamazares et al., 2018; Dho et al., 2018). Clearly, the demand of ^177^Lu is only going to increase and radionuclide generator can complement the current production routes. The long half-life of ^177m^Lu (160.44 days) can potentially lead to on-site and on-demand ^177^Lu supply for a long period of time without the need of weekly irradiations in nuclear reactor (De Vries & Wolterbeek, 2012; Bhardwaj et al., 2017). However, the development of ^177m^Lu/^177^Lu radionuclide generator needs to tackle the great challenge of separating the physically and chemically alike nuclear isomers ^177^Lu and ^177m^Lu.

It has been previously shown that ^177^Lu can be separated from ^177m^Lu due to the chemical effects occurring as a consequence of internal conversion decay of ^177m^Lu (Bhardwaj et al., 2017). Internal conversion decay may result in the emission of multiple Auger electrons, often accompanied with the loss of valence electrons and leaving the atom in a highly positively charged state which can result in bond rupture (Cooper, 1942). This principle presents a possibility to separate two isomers, provided that a separation process that can quickly & selectively capture the freed ions is feasible. Additionally, from a radionuclide generator perspective, the separation process should also allow the periodic extraction of the produced daughter radionuclide during the lifetime of the generator.

Previously, a column chromatography based ^177^Lu-^177m^Lu separation process has been reported, where the ^177m^Lu complexed with DOTATATE has been immobilized on a tC-18 silica and the freed ^177^Lu ions produced after the decay have been separated using a mobile phase flow (Bhardwaj et al., 2017). The ^177^Lu/^177m^Lu activity ratio of 250 has been reached after separation compared to the equilibrium ^177^Lu/^177m^Lu activity ratio of 0.25. However, in order to fulfil the clinical demand the separation method should provide ^177^Lu having minimum breakthrough of ^177m^Lu. The current direct production route delivers ^177^Lu with ^177^Lu/^177m^Lu activity ratio ranging from 4000 to 10,000 (Dvorakova et al., 2008; Pawlak et al., 2004; Knapp FFJA et al., 1995; Das et al., 2007; Chakraborty et al., 2014), while the indirect production route affords the no-carrier added ^177^Lu with almost negligible amount of ^177m^Lu (Watanabe et al., 2015).

In this work, a radionuclide generator for the production of ^177^Lu based on the pair of nuclear isomer^177m^Lu-^177^Lu is presented. The ^177m^Lu-^177^Lu separation has been performed using liquid-liquid extraction (LLE). LLE has been explored several times before in the development of other radionuclide generators, such as ^99^Mo/^99m^Tc, ^68^Ge/^68^Ga, ^188^Re/^188^W, and ^90^Y/^90^Sr radionuclide generators (Le Minh & Lengyel, 1989; Fikrle et al., 2010; Bhatia & Turel, 1989; Boyd, 1982; Ehrhardt & Welch, 1978; Mushtaq et al., 2007; Dutta & Mohapatra, 2013). The present work demonstrates the application of LLE in ^177^Lu-^177m^Lu separation which can potentially lead to a commercial ^177m^Lu/^177^Lu radionuclide generator. The metastable isomer, ^177m^Lu, was complexed with the chelating agents (DOTA and DOTATATE) and the freed ^177^Lu ions was extracted in dihexyl ether using Di-(2-ethylhexyl) phosphoric acid (DEHPA) as the cation extracting agent.

## Materials and methods

### Materials

Lutetium chloride hexahydrate, LuCl_3_.6H_2_O (≥99.99%), di (2-ethylhexyl) phosphoric acid, DEHPA (97%), di-n-hexyl ether, DHE (97%), sodium acetate (≥99%), chelex resin (chelex-100, 50–100 mesh) and acentonitrile (99.3%) were purchased from Sigma Aldrich. 1,4,7,10-tetraazacyclododecane N, N′, N″, N″’-tetraacetic acid, DOTA (98%) was purchased from ABCR GmBH & Co. KG Germany. DOTATATE was obtained as a kind gift from Erasums Medical Centre (Rotterdam) and was produced by Biosynthema, MO, USA. The lutetium-177 (^177^Lu) used in the optimization studies was produced by irradiating around 1 mg of natural LuCl_3_.6H_2_O in the Hoger Onderwijs Reactor Delft (HOR) with a thermal neutron flux of 4.72*10^12^ neutrons∙s^− 1^∙cm^− 2^ (less than 1.5% epithermal contribution) and an irradiation time of 10 h. The solid sample was weighed inside polyethylene capsule and sealed, packed inside polyethylene rabbits. After irradiation, the samples were left for a cooling period of 3 days, resulting in the production of around 17 MBq of ^177^Lu. The capsules were opened and transferred into a plastic vial containing 2.5 mL, pH -3, HCl solution, resulting in a 1 mM [^177^Lu]LuCl_3_ solution.

The Lutetium-177 m (^177m^Lu) source was provided by IDB- Holland as a 1 mM [^177m^Lu]LuCl_3_ solution with about 5 MBq ^177m^Lu per g of solution.

### Methods

#### γ ray spectroscopy analysis

All the activity measurements were performed on a well-type HPGe detector for counting time up to 5 h to reduce the error from the counting statistics to less than 5%. The measurement of the samples obtained at the end of LLE was repeated after 3–4 half-lives of ^177^Lu to decrease the background and measure the ^177m^Lu activity with less than 5% uncertainty. The efficiency calibration for different peaks was performed using a known activity of ^177^Lu source supplied by IDB Holland. The obtained gamma ray spectra were analysed using an in-house software to calculate the activity of each fraction (Blaauw, 1993). In order to minimize the error, all the vials were weighed before and after the fraction collection.

#### Preparation of aqueous phase

The ^177m^Lu containing LuCl_3_ solution (1 mM) was used to prepare [^177m^Lu]Lu-DOTA complex in three different molar ratios (1:1, 1:2, 1:4). Typically, 1 mM [^177m^Lu]LuCl_3_ solution (0.150 mL, 0.150 μmoles) was mixed with 0.01 M DOTA in different molar ratios (1:1, 1:2 & 1:4) in the presence of 0.150 mL, 1 M sodium acetate- acetic acid buffer at pH 4.3. The reaction mixture was heated at 80 °C for 30 min. The [^177m^Lu]Lu-DOTATATE complex was synthesized as reported previously in a Lu:DOTATATE molar ratio of 1:4 (Bhardwaj et al., 2017). Typically, 1 mM [^177m^Lu]LuCl_3_ solution (0.050 mL, 0.050 μmoles) was mixed with 0.200 μmol DOTATATE solution in the presence of 0.150 mL, 1 M sodium acetate- acetic acid buffer (pH- 4.3). The reaction mixture was heated at 80 °C for about 1 h followed by incubation at room temperature for about 1 h.

The complex formation was confirmed using instant thin layer chromatography. Free ^177m^Lu ions traces were removed using a cation exchange resin (chelex-100). (Details in S1, Additional file 1).

#### Liquid-liquid extraction (LLE) procedure

**Fig. 1:**
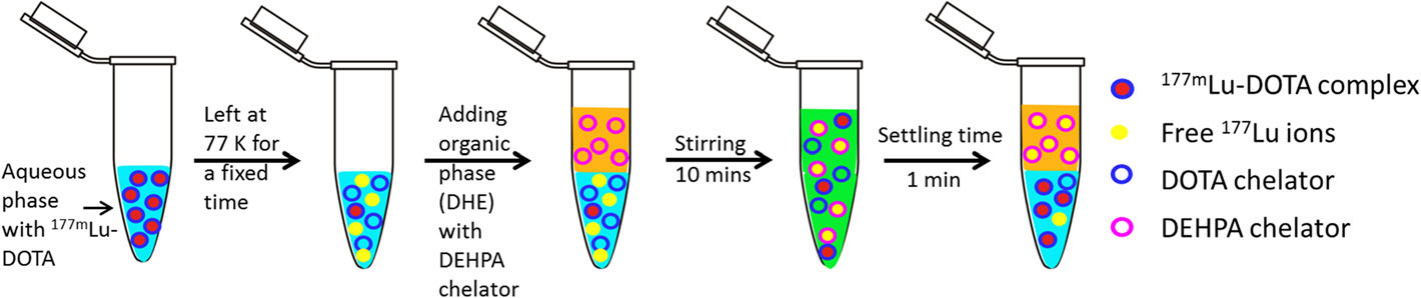
Schematic representation of liquid-liquid extraction to extract the freed ^177^Lu ions

The schematic representation of LLE to separate the freed ^177^Lu ions from the complexed ^177m^Lu ions is shown in Fig. 1.

All the LLE experiments were performed in 2 mL Eppendorf by placing them in a shaking incubator at room temperature. The aqueous and the organic phases were mixed in volumetric ratio (1:1) at 1400 rpm for a stirring time of about 10 min. The stirring time of 10 min was optimised by studying the ^177^Lu extraction efficiency as a function of extraction time (see Additional file 1 Figure S1(b), S2, supplementary information). At the end of stirring, the layer separation was achieved after a settling time of about one minute. In order to avoid any contamination of the aqueous layer in the organic layer, only the upper 2/3rd organic layer was taken out using a 20- 200uL pipette in all the LLE experiments. The pipetted organic layer was transferred to a pre-weighed vial to know the exact amount of organic phase removed in each extraction.

First, free ^177^Lu cations were extracted from a 0.3 mL, pH -4, 1 mM [^177^Lu]LuCl_3_ solution as the aqueous phase. The organic phase consists of 0.3 mL dihexyl ether containing different DEHPA concentrations, namely 0.01, 0.05, 0.1, 0.15, 0.2, 0.4, 0.6, 1.0, 1.2 and 1.6 M. At the end of LLE, the ^177^Lu activity in the organic and the aqueous layer was measured using γ ray spectroscopy to obtain the ^177^Lu extraction efficiency (EE). The EE is defined as the percentage of the ^177^Lu activity moving from the aqueous phase in to the organic phase after the extraction. All the experiments were performed in triplicate.

Subsequently, the LLE was performed to extract the freed ^177^Lu ions from the aqueous phase containing [^177m^Lu]Lu-DOTATATE, [^177m^Lu]Lu-DOTA complex. For [^177m^Lu]Lu-DOTATATE complex, the ^177^Lu extraction was performed successively at varying ^177^Lu accumulation periods for a total time period of up to 60 days. For, [^177m^Lu]Lu-DOTA complex, the freed ^177^Lu ions were extracted successively at every 7 days for a total time period of 35 days. In between the extractions, the [^177m^Lu]Lu-DOTA and [^177m^Lu]Lu-DOTATATE complexes were left in a liquid N_2_ tank to allow for the accumulation of freed ^177^Lu ions. The ^177^Lu separation was performed by bringing the vial out of the liquid N_2_ tank and quickly adding the 0.01 M DEHPA in DHE in a 1:1 volumetric ratio (0.3 mL: 0.3 mL), at room temperature and 10 min of stirring time, as shown schematically in Fig. 1. At the end of LLE, the ^177^Lu and ^177m^Lu activity in the organic layer was measured using γ ray spectroscopy to calculate the amount of ^177^Lu and ^177m^Lu ions extracted in the organic phase and the ^177^Lu/^177m^Lu activity ratio.

The ^177^Lu extraction efficiency is defined as the amount of ^177^Lu ions that were extracted into the organic phase divided by the theoretically produced ^177^Lu ions (see section S3, eq. S2 in Supplementary Information). The percentage of ^177m^Lu extracted is defined as the activity of ^177m^Lu ions measured in organic phase after the LLE divided by the starting activity of the ^177m^Lu ions in the aqueous phase.

## Results

### ^177^Lu/ ^177m^Lu separation using [^177m^Lu]Lu-DOTATATE complex

**Fig. 2:**
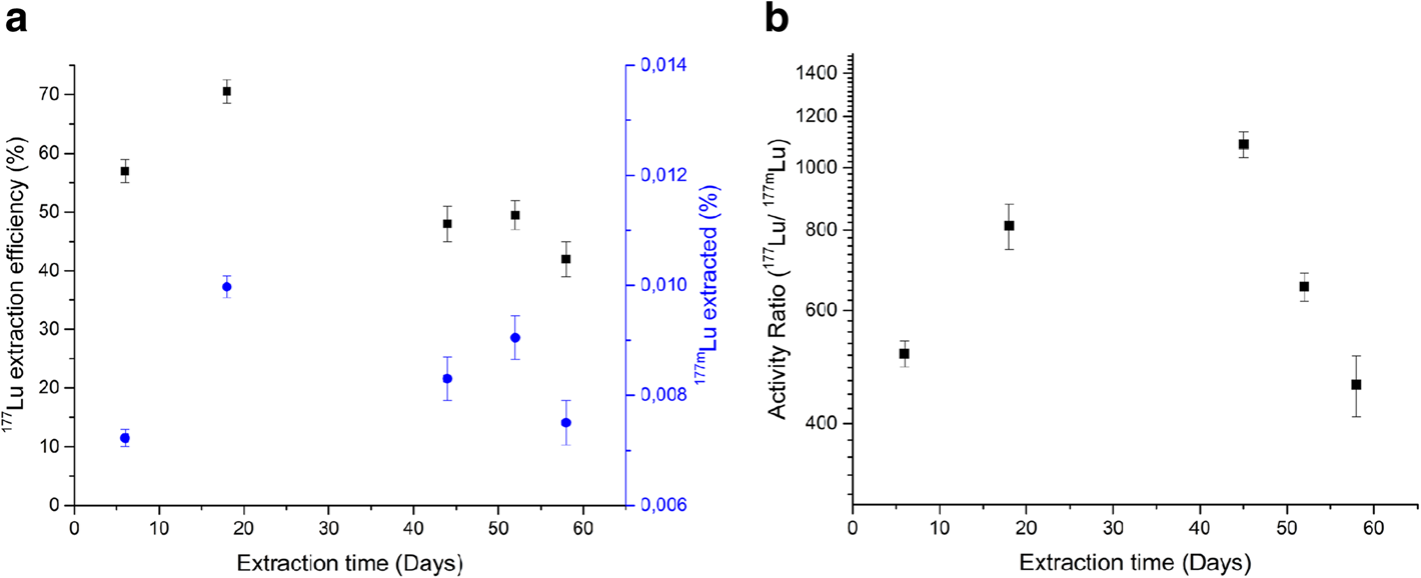
**a** The ^**177**^Lu extraction efficiency (y axis, left) and the % ^177m^Lu extracted (y axis, right) at different extraction time during the successive LLE of free ^**177**^Lu ions from [^177m^Lu]Lu-DOTATATE complex using 0.01 M DHEPA in DHE. **b** The ^**177**^Lu/^177m^Lu activity ratio obtained in the organic phase at different extraction time. The error bars represent the error in the individual measurements due to counting statistics

The ^177^Lu/ ^177m^Lu separation was performed using [^177m^Lu]Lu-DOTATATE complex synthesized in the presence of an excess of DOTATATE (Lu:DOTATATE molar ratio of 1:4). The ^177^Lu ions production via the decay of ^177m^Lu is represented by eq. S1, Supplementary Information, S3 and the expected growth of ^177^Lu ions with the increase in the ^177^Lu accumulation period is shown in Additional file 1 Figure S2, Supplementary Information. The amount of ^177^Lu ions produced increases with an increase in ^177^Lu accumulation period and reaches a maximum after 32 days of ^177^Lu accumulation. In the presented results, the freed ^177^Lu ions were extracted from [^177m^Lu]Lu-DOTATATE complex by performing LLE successively after different ^177^Lu accumulation intervals. Figure 2 (a)&(b) show the ^177^Lu extraction efficiency and percentage of the ^177m^Lu ions extracted in the organic phase at the end of the LLE at different time intervals, respectively. An average ^177^Lu extraction efficiency of 60 ± 10% was obtained at the end of LLE. This is 40% less than the 99 ± 2% ^177^Lu extraction efficiency observed during the LLE of ^177^Lu ions from a 1 mM [^177^Lu]LuCl_3_ solution using 0.01 M DEHPA in DHE (see Additional file 1 Figure S1, supplementary information S2). Additionally, along with the ^177^Lu ions, 0.0085 ± 0.0015% of the starting ^177m^Lu activity was also extracted in the organic phase. Figure 2(b), shows the ^177^Lu/^177m^Lu activity ratios obtained after different extractions. An increase in the ^177^Lu/^177m^Lu activity ratio is observed with an increase in the time interval between the extractions. The maximum ^177^Lu/^177m^Lu activity ratio of 1086 ± 40 is obtained during the LLE at 43 days after a ^177^Lu accumulation period of 26 days. A decrease in the ^177^Lu accumulation period leads to a decrease in the ^177^Lu/ ^177m^Lu activity ratios. The ^177^Lu/^177m^Lu activity ratios 600 ± 100 was obtained for ^177^Lu accumulation periods between 6 and 10 days.

### ^177^Lu/^177m^Lu radionuclide separation using [^177m^Lu]Lu-DOTA complex

**Fig. 3:**
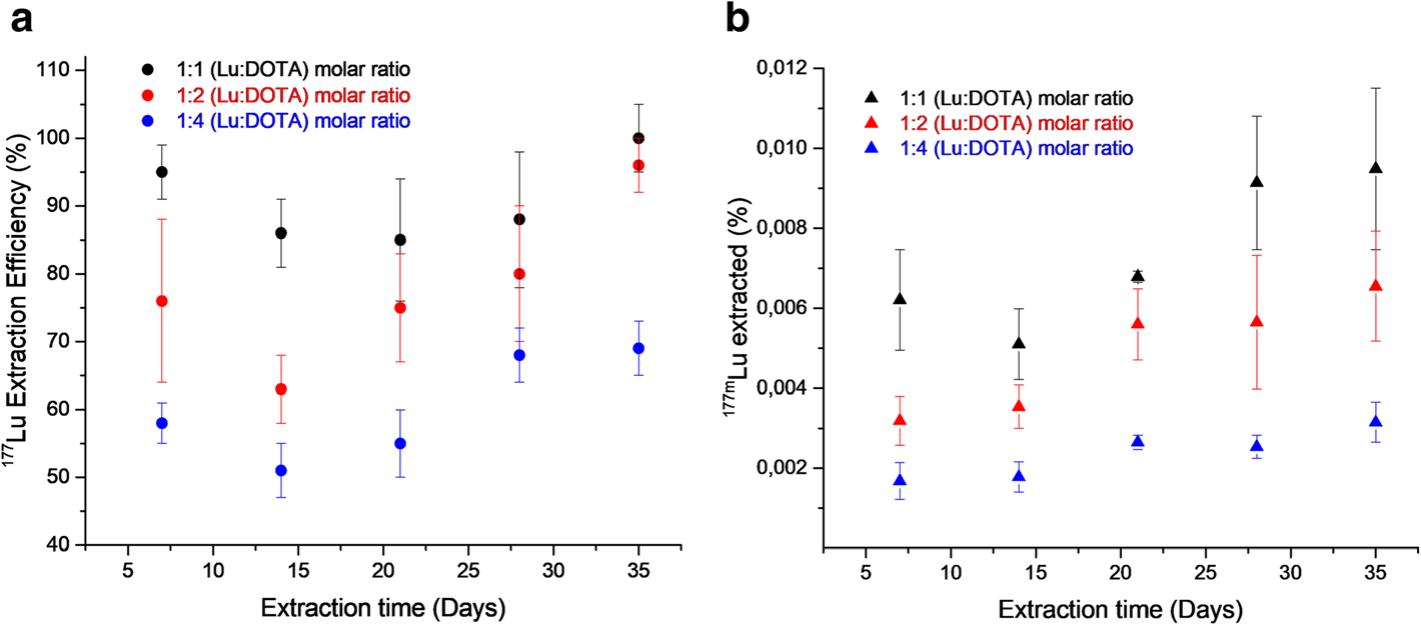
**a** The ^**177**^Lu extraction efficiency and **b** the percent ^177m^Lu extracted at different extraction time during the successive LLE of free ^**177**^Lu ions from [^177m^Lu]Lu-DOTA complex using 0.01 M DHEPA in DHE. The experiments were performed for three different Lu: DOTA molar ratios, **(1:1)** in **black**, **(1:2)** in **red** and **(1:4)** in **blue**. The data points represent the average ± STD of three experiments, the individual error in measurements due to counting statistics is less than 5%

**Fig. 4:**
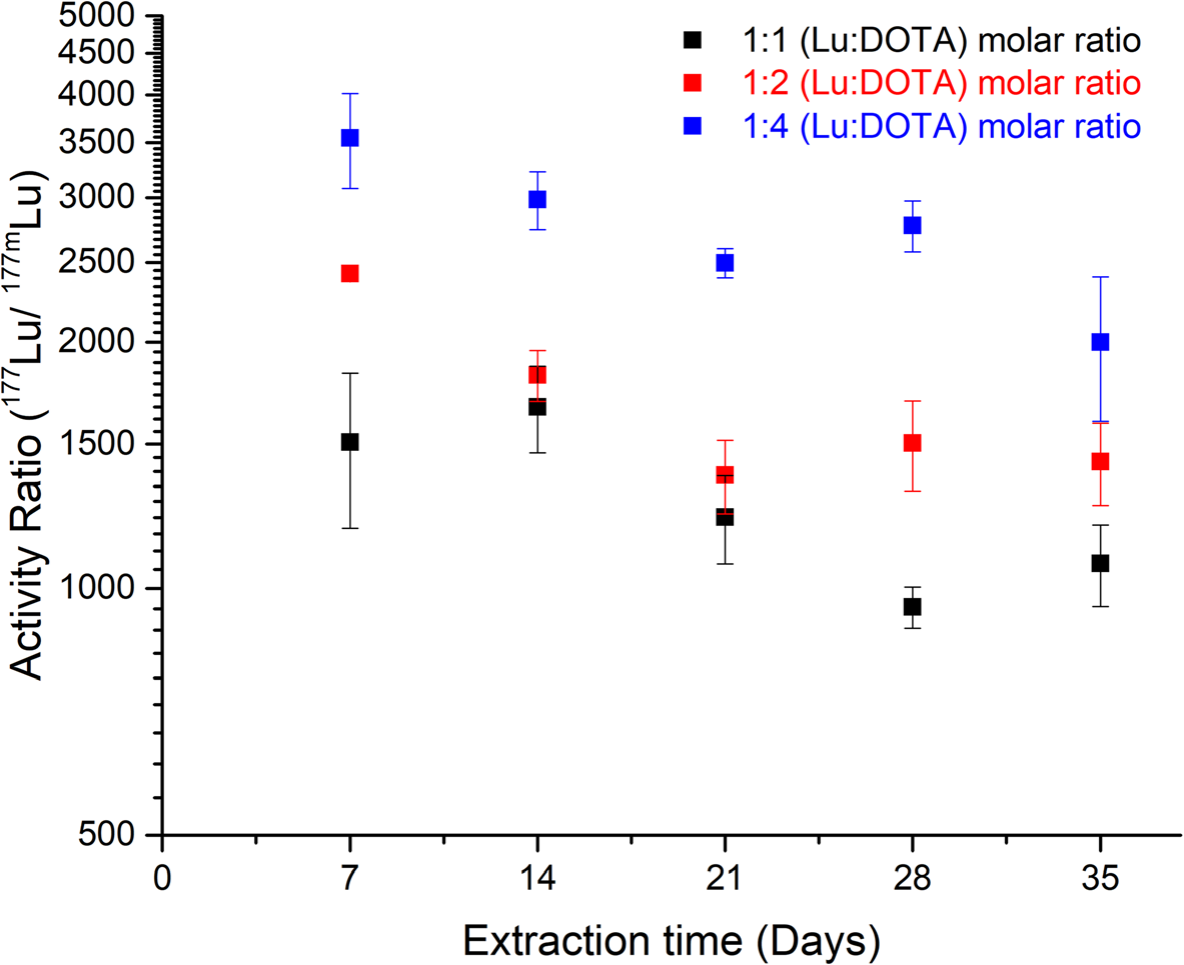
The ^**177**^Lu/ ^177m^Lu activity ratio obtained during the successive LLE of free ^**177**^Lu ions from the [^177m^Lu]Lu-DOTA complex. The experiments were performed with three Lu: DOTA molar ratios, **(1:1)** in **black**, **(1:2)** in **red** and **(1:4)** in **blue**. The data points represent the average ± STD of three experiments, the individual error in measurements due to counting statistics is less than 5%

The results obtained when the LLE was performed to extract the freed ^177^Lu ions from the [^177m^Lu]Lu-DOTA complex are shown in Fig. 3&4. The LLE was performed successively at time intervals of 7 days. Figure 3(a) shows the effect of Lu: DOTA molar ratios on ^177^Lu extraction efficiency. Figure 3(b) displays the percentage of initial ^177m^Lu activity extracted in the organic phase at the end of LLE for the different Lu: DOTA molar ratios.

It can be seen from Fig. 3(a) that the ^177^Lu extraction efficiency reaches a maximum value of 95 **±** 4% when Lu & DOTA were present in 1:1 M ratio and decreases to 58 **±** 2% for 1:4 Lu:DOTA molar ratio. Further, the ^177^Lu extraction efficiency remains almost constant for the first three extractions followed by a slight increase during the 4th and 5th extraction for all the three Lu:DOTA molar ratios. Figure 3(b) shows that 0.0061 **±** 0.0015% of ^177m^Lu activity was extracted in the first extraction when Lu and DOTA were present in 1:1 M ratio, which got reduced to 0.0020 **±** 0.0010% for the Lu:DOTA molar ratio 1:4. The percentage of ^177m^Lu activity extracted remains almost constant during the successive extractions in the presence of excess DOTA, and increases from 0.0061 **±** 0.0015% to 0.0095 **±** 0.0015% in the presence of 1:1 Lu:DOTA molar ratio. The error bars in Fig. 3 represent the standard deviation in the results of three experiments performed in parallel.

Figure 4 shows the ^177^Lu/^177m^Lu activity ratios observed in the organic phase at the end of LLE for the three different Lu:DOTA molar ratios. It reveals that the ^177^Lu/^177m^Lu activity ratio increases with an increase in the molar quantities of DOTA. The highest ^177^Lu/^177m^Lu activity ratio of 3500 ± 500 was obtained when DOTA was present in excess (1:4) and decreases to around 1500 ± 600 in the presence of 1:1 Lu:DOTA molar ratio. Further, a slight decrease in the ^177^Lu/^177m^Lu activity ratios was observed in every successive LLE performed during the 35 days of experiments. The fifth ^177^Lu extraction performed at the end of the experiments resulted in a 40 ± 5% decrease in the ^177^Lu/^177m^Lu activity ratios compared to the ^177^Lu/^177m^Lu activity ratio obtained in the first extraction.

Overall, the ^177^Lu/^177m^Lu activity ratios obtained using DOTA as chelating agent were about 5 times higher when compared with ^177^Lu/^177m^Lu activity ratios obtained using DOTATATE for a ^177^Lu accumulation period of around 7 days. Also, the percentage of ^177m^Lu activity extracted in the organic phase was about 5 times higher with DOTATATE than that observed with DOTA as the ^177m^Lu complexing agent.

## Discussion

The separation of the isomers ^177^Lu and ^177m^Lu based on the nuclear decay after effects is achieved using liquid-liquid extraction (LLE) as the separation method and the [^177m^Lu]Lu-DOTA, [^177m^Lu]Lu-DOTATATE complexes. The ^177^Lu production at 77 K resulted in negligible dissociation of the starting [^177m^Lu]Lu-DOTA based complexes, and increases the quality of extracted ^177^Lu remarkably. The freed ^177^Lu ions were extracted in the organic phase by performing the LLE at room temperature. The separation was done sufficiently fast resulting in production of limited quantities of free ^177m^Lu ions.

In the present work, the ^177^Lu/^177m^Lu activity ratio of 1086 ± 40 is achieved using [^177m^Lu]Lu-DOTATATE complex which is about 4 times higher than the previously reported ^177^Lu/ ^177m^Lu activity ratio of 250 realized using the same [^177m^Lu]Lu-DOTATATE complex (Bhardwaj et al., 2017). In the previously reported method, the ^177^Lu ion accumulation was performed at 10 °C and the temperature could not be decreased further because of experimental limitations. In contrast, the present LLE based separation allows the ^177^Lu accumulation at 77 K. At 77 K, the rate constants for the chemical reactions (i.e. association-dissociation kinetics) are extremely low making the ^177m^Lu contribution coming from the dissociation of the [^177m^Lu]Lu-DOTATATE complex negligible during the ^177^Lu accumulation period. The ^177m^Lu contribution observed in the present work can be accounted to the dissociation of the [^177m^Lu]Lu-DOTATATE complex during the LLE at room temperature. After the dissociation, the ^177m^Lu and ^177^Lu ions are indistinguishable and they will go into the organic phase with equal rate.

The LLE of ^177^Lu ions from [^177m^Lu]Lu-DOTATATE complex resulted in co-extraction of 0.0085 ± 0.0015% of initial ^177m^Lu activity in the organic phase. This leads to an estimated dissociation rate constant of 1.3*10^− 7^ ± 0.3*10^− 7^ s^− 1^. For Lu-DOTATATE complex, a dissociation constant rate 2*10^− 8^ s^− 1^ has been reported at pH -4.3 and 20 °C (van der Meer et al., 2013). It has also been shown that the Lu-DOTATATE complex is accompanied by the presence of short-lived unstable, mono- and di-protonated (MHL, MH_2_L) complex species (van der Meer et al., 2013). These species have a dissociation rate constant of 8*10^− 5^ s^− 1^ (MHL) & 2*10^− 4^ s^− 1^ (MH_2_L) at pH -4.3 and 20 °C (van der Meer et al., 2013). Therefore, the presently estimated dissociation rate constant does not represent the dissociation of single species, but is rather a combination of the dissociation contribution from three different species i.e. ML, MHL, & MH_2_L. Overall, the [^177m^Lu]Lu-DOTATATE complex behavior clearly highlights the fact that a careful consideration of all the possible species at a certain pH should be given while assessing the role of any complexing agent in ^177^Lu-^177m^Lu separation.

The ^177^Lu/^177m^Lu activity ratio obtained during the LLE of ^177^Lu ions from [^177m^Lu]Lu-DOTATATE complex was found to be influenced by the length of the ^177^Lu accumulation period. The highest ^177^Lu/^177m^Lu activity ratio of 1086 ± 40 was obtained after ^177^Lu accumulation period of 26 days and decreased to 600 ± 200 for accumulation periods of 5 to 10 days. This was expected as the amount of ^177^Lu ions produced from the internal conversion of ^177m^Lu ions grows as the ^177^Lu accumulation period increases. In contrast, the ^177m^Lu contribution is only due to dissociation of the complex taking place during the extraction. Additionally, a ^177^Lu extraction efficiency of 60 ± 10% was observed which can be associated to the loss of free ^177^Lu ions due to their re-association with the excess complexing agent, as reported before by Bhardwaj et al. (Bhardwaj et al., 2017).

The crucial role of association kinetics on ^177^Lu- ^177m^Lu separation is further emphasised by studying the ^177^Lu-^177m^Lu separation in the presence of varying amounts of DOTA as the complexing agent. The ^177^Lu extraction efficiency obtained during the LLE of freed ^177^Lu ions was affected by the applied ratio of complexing agent. The ^177^Lu extraction efficiency of 58 ± 2% was achieved in the presence of excess DOTA (Lu:DOTA molar ratio, 1:4), and it increases to 95 ± 4% when Lu:DOTA was present in the molar ratio 1:1, confirming that the association kinetics of freed ^177^Lu and the excess of DOTA play an important role in the process. Similarly, the extracted ^177m^Lu activity decreases from 0.0060 ± 0.0015% to 0.0020 ± 0.0010% with the increase in the Lu:DOTA molar ratios from (1:1) to (1:4) respectively, due to the re-association of ^177m^Lu ions with the excess of DOTA.

The ^177^Lu/^177m^Lu activity ratios obtained during the LLE of ^177^Lu ions from [^177m^Lu]Lu-DOTA complex were also found to be effected by the starting Lu:DOTA molar ratio. A ^177^Lu/^177m^Lu activity ratio up to 3500 ± 500 was achieved when the LLE was performed using aqueous [^177m^Lu] LuDOTA complex with Lu:DOTA present in the molar ratio 1:4. Remarkably, the obtained ^177^Lu/^177m^Lu activity ratios are very close to the ^177^Lu/^177m^Lu activity ratios of 4000–10,000 associated to the direct-route production of ^177^Lu supplied to the clinics (Das et al., 2007; Chakraborty et al., 2014). These ratios were found to decrease with the decrease in the amount of DOTA, i.e. an activity ratio of 1500 ± 600 was observed when Lu and DOTA were present in the molar ratio 1:1. The presence of excess DOTA leads to a proportional decrease in the amount of both ^177^Lu and ^177m^Lu ions due to re-association. However, the ^177^Lu production from internal conversion of ^177m^Lu ions adds to a constant positive contribution in the amount of ^177^Lu ions, which leads to an overall increase in the ^177^Lu/^177m^Lu activity ratios.

Finally, the observed decrease in the ^177^Lu/^177m^Lu activity ratio with the increase in time are well in agreement with the theoretically expected ratios based on the ^177m^Lu and ^177^Lu extracted shown in Fig. 3 and incorporating the effect of incomplete organic phase removal on every successive extraction (see Additional file 1 Figure S3, supplementary information). The reported separation method suffers from the drawback of incomplete organic phase removal during the LLE. The residual 1/3rd of the organic phase left unrecovered after every LLE contains un-extracted ^177^Lu and ^177m^Lu ions. The ^177^Lu ions will reduce to about a half after accumulation time of 7 days, but the ^177m^Lu ions will remain almost unchanged as they have a half-life of 160.44 days. They will add to the total amount of free ^177m^Lu ions in the successive extraction and correspondingly to a decrease the ^177^Lu/ ^177m^Lu activity ratio. In case of a complete organic phase removal, the separation method could lead to a constant value of ^177^Lu/^177m^Lu activity ratio of around 3500 on performing periodic ^177^Lu extraction every 7 days. Additionally, the use of longer ^177^Lu accumulation period of 32 days will lead to 1.7 times more ^177^Lu production compared to 7 days ^177^Lu accumulation period. This can potentially lead to an activity ratio of 7000 on considering a constant 0.0020 ± 0.0010% ^177m^Lu contribution due to dissociation and 58 ± 2% ^177^Lu extraction efficiency. In such a case, the extracted ^177^Lu would contain a ^177m^Lu contribution as low as 0.01% and would be comparable to the direct route ^177^Lu production.

It should be pointed that the specific activity of the produced ^177^Lu is not a discussed parameter since the starting ^177m^Lu source has very low specific activity and therefore also the extracted ^177^Lu. Consequently, the values would not represent a fair comparison with the commercially available ^177^Lu. Additionally, the extracted ^177^Lu ions have not been stripped from the organic phase back into the aqueous phase considering that it is a well-reported process in literature (Trtic-Petrovic et al., 2010).

Overall, the presented work is an important milestone towards the development of a ^177m^Lu/^177^Lu radionuclide generator for clinical application. It also establishes the possibility of employing other separation techniques such as micro-fluidic separation (Davide et al., 2014), membrane based liquid-liquid extraction (Pedersen-Bjergaard & Rasmussen, 2008) or an automatized LLE separation devices that can allow the commercialization of LLE based ^177m^Lu/ ^177^Lu radionuclide generator. However, there are several aspects that needs further investigation and optimization. Firstly, the back extraction of ^177^Lu from the organic phase and the complete removal of any traces of organic solvents will be crucial for its potential commercialization. Secondly, this work has been performed at lab-scale with low activity levels and excludes the effect of radiolysis on the proposed ^177m^Lu-^177^Lu separation method. The radiolysis can impact the quality of the produced ^177^Lu and should be carefully evaluated in the future investigations. Lastly, the described method can be further optimized in terms of shorter extraction time, use of lower temperature to perform the ^177^Lu extraction improve the produced ^177^Lu quality.

## Conclusion

A novel ^177m^Lu-^177^Lu separation method is developed that allows the ^177^Lu production via internal conversion of ^177m^Lu at low temperatures (77 K) and the use of ultra-stable ^177m^Lu complexes with liquid-liquid extraction. For the best conditions, the [^177m^Lu]Lu-DOTA complex and LLE provides a ^177^Lu/^177m^Lu activity ratio of 3500 ± 500. A value that is close to the ^177^Lu/^177m^Lu activity ratio 4000–10,000 obtained during the ^177^Lu production via the direct route and exemplifies the potential applicability of the ^177m^Lu/^177^Lu generator in clinical studies. Future research will be focused on further optimization of novel ^177^Lu-^177m^Lu separation technologies aimed to ultimately lead to a clinically applicable ^177m^Lu/^177^Lu radionuclide generator. The around the clock availability of ^177^Lu via a ^177m^Lu/ ^177^Lu radionuclide generator can significantly accelerate the research on ^177^Lu based radiopharmaceuticals and help in realizing its full potential in nuclear medicine.

## Additional file

Additional file 1: **Figure S1.** The ^177^Lu extraction efficiency of 0.3mL, 1mM [^177^Lu]LuCl_3_ as a function of a) varying DEHPA concentration in dihexylether and b) as a function of phase stirring time. Data points represent the average and standard deviation for six experiments. **Figure S2.** The amount of ^177^Lu produced from 1 MBq of ^177m^Lu for different ^177^Lu accumulation period as calculated by using equation 1. **Figure S3.** The ^177^Lu/^177m^Lu activity ratio obtained at different elution time when the LLE is performed with [^177m^Lu]Lu-DOTA complex synthesized in a molar ratio 1:4. The data points represent the experimentally observed ratios, while the dotted line represents the expected activity ratios with 60% ^177^Lu extraction efficiency and 0.002% ^177m^Lu ions leakage. (DOCX 87 kb)

## Data Availability

All data generated or analyzed during this study are included in this published article [and its supplementary information files].

## Abbreviations

DEHPA: di (2-ethylhexyl) phosphoric acid
DHE: dihexyl ether
DOTA: 1,4,7,10-tetraazacyclododecane N, N′, N″, N″’-tetraacetic acid
DOTATATE: DOTA-(Tyr^3^)-octreotate
LLE: Liquid Liquid Extraction

## References

[CR1] Banerjee S, Pillai MR, Knapp FF (2015). Lutetium-177 therapeutic radiopharmaceuticals: linking chemistry, radiochemistry, and practical applications. Chem Rev.

[CR2] Bhardwaj R, van der Meer A, Das SK, de Bruin M, Gascon J, Wolterbeek HT (2017). Separation of nuclear isomers for cancer therapeutic radionuclides based on nuclear decay after-effects. Sci Rep.

[CR3] Bhatia DS, Turel ZR (1989). Solvent extraction of99mTc/VII/ with methylene blue into nitrobenzene. J Radioanal Nucl Chem.

[CR4] Blaauw M. The holistic analysis of gamma-ray spectra in instrumental neutron activation analysis; 1993.

[CR5] Boyd RE (1982). Technetium-99m generators—the available options. The International Journal of Applied Radiation and Isotopes.

[CR6] Chakraborty S, Vimalnath KV, Lohar S, Shetty P, Dash A (2014). On the practical aspects of large-scale production of 177Lu for peptide receptor radionuclide therapy using direct neutron activation of 176Lu in a medium flux research reactor: the Indian experience. J Radioanal Nucl Chem.

[CR7] Cooper EP (1942). On the separation of nuclear isomers. Phys Rev.

[CR8] Das T, Banerjee S (2016). Theranostic applications of Lutetium-177 in radionuclide therapy. Curr Radiopharm.

[CR9] Das T, Chakraborty S, Banerjee S, Venkatesh M (2007). On the preparation of a therapeutic dose of 177Lu-labeled DOTA–TATE using indigenously produced 177Lu in medium flux reactor. Appl Radiat Isot.

[CR10] Das T, Pillai MR (2013). Options to meet the future global demand of radionuclides for radionuclide therapy. Nucl Med Biol.

[CR11] Davide C, PJ M, SG W (2014). The use of microfluidic devices in solvent extraction. Journal of Chemical Technology & Biotechnology.

[CR12] De Vries DJ, Wolterbeek H (2012). The production of medicinal 177Lu and the story of 177mLu: detrimental by-product or future friend?. Tijdschr Nucl Geneeskd.

[CR13] Dho SH, Kim SY, Chung C, Cho EH, Lee S-Y, Kim JY (2018). Development of a radionuclide-labeled monoclonal anti-CD55 antibody with theranostic potential in pleural metastatic lung cancer. Sci Rep.

[CR14] Dutta S, Mohapatra PK (2013). Studies on the separation of 90Y from 90Sr by solvent extraction and supported liquid membrane using TODGA: role of organic diluent. J Radioanal Nucl Chem.

[CR15] Dvorakova Z, Henkelmann R, Lin X, Türler A, Gerstenberg H (2008). Production of 177Lu at the new research reactor FRM-II: irradiation yield of 176Lu(n,γ)177Lu. Appl Radiat Isot.

[CR16] Ehrhardt GJ, Welch MJ (1978). Journal of nuclear medicine: official publication. Society of Nuclear Medicine.

[CR17] Emmett L, Willowson K, Violet J, Shin J, Blanksby A, Lee J (2017). Lutetium 177 PSMA radionuclide therapy for men with prostate cancer: a review of the current literature and discussion of practical aspects of therapy. Journal of Medical Radiation Sciences.

[CR18] Fikrle M, Kučera J, Šebesta F (2010). Preparation of 95mTc radiotracer. J Radioanal Nucl Chem.

[CR19] Hofman MS, Violet J, Hicks RJ, Ferdinandus J, Thang SP, Akhurst T (2018). PSMA-617 radionuclide treatment in patients with metastatic castration-resistant prostate cancer (LuPSMA trial): a single-Centre, single-arm, phase 2 study. The Lancet Oncology.

[CR20] https://wayback.archive-it.org/7993/20180725092236/https://www.fda.gov/Drugs/DevelopmentApprovalProcess/DrugInnovation/ucm592464.htm.

[CR21] Knapp FF, Baum RP (2012). Radionuclide Generators; A New Renaissance in the Development of Technologies to Provide Diagnostic and Therapeutic Radioisotopes for Clinical Applications. Curr Radiopharm.

[CR22] Knapp FF, Dash A (2016). Radionuclide generator systems represent convenient production systems to provide therapeutic radionuclides. Radiopharmaceuticals for therapy.

[CR23] Knapp FF, Mirzadeh S (1994). The continuing important role of radionuclide generator systems for nuclear medicine. Eur J Nucl Med.

[CR24] Knapp FF, Pillai MRA, Osso JA, Dash A (2014). Re-emergence of the important role of radionuclide generators to provide diagnostic and therapeutic radionuclides to meet future research and clinical demands. J Radioanal Nucl Chem.

[CR25] Knapp FFJA, K.R.; Beets, A.L.; Luo, H.; McPherson, D.W. & Mirzadeh, S. Nuclear medicine program progress report for quarter ending September 30, 1995, report,.

[CR26] Le Minh T, Lengyel T (1989). On the separation of molybdenum and technetium crown ether as extraction agent. J Radioanal Nucl Chem.

[CR27] Mushtaq A, Bukhari Tanveer H, Khan Islam U. Extraction of medically interesting 188Re-perrhenate in methyl ethyl ketone for concentration purposes. Radiochim Acta. 2007:535.

[CR28] Pawlak D, Parus JL, Sasinowska I, Mikolajczak R (2004). Determination of elemental and radionuclidic impurities in 177Lu used for labeling of radiopharmaceuticals. J Radioanal Nucl Chem.

[CR29] Pedersen-Bjergaard S, Rasmussen KE (2008). Liquid-phase microextraction with porous hollow fibers, a miniaturized and highly flexible format for liquid–liquid extraction. J Chromatogr A.

[CR30] Pillai MRA, Ashutosh D, Knapp FF (2012). Rhenium-188: availability from the 188W/188Re generator and status of current applications. Curr Radiopharm.

[CR31] Repetto-Llamazares Ada H. V., Malenge Marion M., O'Shea Adam, Eiríksdóttir Bergthóra, Stokke Trond, Larsen Roy H., Dahle Jostein (2018). Combination of 177 Lu-lilotomab with rituximab significantly improves the therapeutic outcome in preclinical models of non-Hodgkin's lymphoma. European Journal of Haematology.

[CR32] Roesch F, Riss PJ (2010). The renaissance of the 68Ge/68Ga radionuclide generator initiates new developments in 68Ga radiopharmaceutical chemistry. Curr Top Med Chem.

[CR33] Trtic-Petrovic TM, Kumric KR, Dordevic JS, Vladisavljevic GT (2010). Extraction of lutetium(III) from aqueous solutions by employing a single fibre-supported liquid membrane. J Sep Sci.

[CR34] van der Meer A, Breeman WAP, Wolterbeek B (2013). Reversed phase free ion selective radiotracer extraction (RP-FISRE): a new tool to assess the dynamic stabilities of metal (−organic) complexes, for complex half-lives spanning six orders of magnitude. Appl Radiat Isot.

[CR35] Watanabe S, Hashimoto K, Watanabe S, Iida Y, Hanaoka H, Endo K (2015). Production of highly purified no-carrier-added 177Lu for radioimmunotherapy. J Radioanal Nucl Chem.

