# Supplementary material for: Radionuclide generator-based production of therapeutic 177Lu from its long-lived isomer 177mLu
Source: EJNMMI Radiopharm Chem. 2019 Jul 15;4:13. doi: 10.1186/s41181-019-0064-5 (PMC6629729; doi:10.1186/s41181-019-0064-5)
Supplement: Supplementary file 1 — Figure S1. The 177Lu extraction efficiency of 0.3mL, 1mM [177Lu]LuCl3 as a function of a) varying DEHPA concentration in dihexylether and b) as a function of phase stirring time. Data points represent the average and standard deviation for six experiments. Figure S2. The amount of 177Lu produced from 1 MBq of 177mLu for different 177Lu accumulation period as calculated by using equation 1. Figure S3. The 177Lu/177mLu activity ratio obtained at different elution time when the LLE is performed with [177mLu]Lu-DOTA complex synthesized in a molar ratio 1:4. The data points represent the experimentally observed ratios, while the dotted line represents the expected activity ratios with 60% 177Lu extraction efficiency and 0.002% 177mLu ions leakage. (DOCX 87 kb) [file 41181_2019_64_MOESM1_ESM.docx]

**Supplementary information**

**Radionuclide generator-based production of therapeutic ^177^Lu from its long-lived isomer ^177m^Lu**

Rupali Bhardwaj ^1, 2^**,** Hubert Th. Wolterbeek ^1^, Antonia G. Denkova ^1^, Pablo Serra-Crespo ^1, *^

**^1^**Radiation and Isotopes for Health, Department of Radiation Science and Technology, Faculty of Applied Sciences, Technical University Delft, Mekelweg 15, 2629 JB, Delft, The Netherlands

^2^Catalysis Engineering , Department of Chemical Engineering, Faculty of Applied Sciences, Delft University of Technology, Van der Maasweg 9, 2629 HZ, Delft, The Netherlands

Corresponding Author: Pablo Serra-Crespo, email-[.P.SerraCrespo@tudelft.nl](mailto:.P.SerraCrespo@tudelft.nl). Room: 2.00.380, Mekelweg 15, 2629 JB Delft, The Netherlands. Phone number: +31 15 27 85309

# Thin layer chromatography and cleaning of the ^177m^Lu complexes with chelex resin.

At the end of synthesis, the product formation was confirmed using instant thin layer chromatography using silica plate as the stationary phase and acetonitrile: water (1:4) as the mobile phase. The uncomplexed ^177m^Lu ions stayed at the bottom (Rf = 0) while the complex moves to the top with the mobile phase ([^177m^Lu]Lu-DOTA with a Rf = 9 and [^177m^Lu]Lu-DOTA with a Rf = 5). The complexation yields > 99% was obtained, and any trace of the free ^177m^Lu ions were removed by passing the complexes 2- 3 times through activated chelex resin at pH 4.3.

The [^177m^Lu]Lu-DOTA and [^177m^Lu]Lu-DOTATATE complexes are passed through activated chelex resin to remove any free un-complexed Lutetium ions. The chelex resin was activated by washing with water (2- 3 times) and 0.1M sodium acetate-acetic acid buffer, pH 4.3 (2- 3 times). The activated resin and the synthesized Lu complex were left stirring together at 20°C for about 10 minutes. At the end of 30 minutes, the aqueous complex was pipetted out using a 20-200 µL pipette. The aqueous complex was then transferred in a pre-weighed vial, and a small aliquot was used to measure the initial ^177m^Lu activity.

# ^177^Lu extraction efficiency as a function of time and DEHPA concentration.

|  |  |
| --- | --- |

Figure S1: The ^177^Lu extraction efficiency of 0.3mL, 1mM [^177^Lu]LuCl_3_ as a function of varying DEHPA concentration in dihexylether (a) and as a function of phase stirring time (b). Data points represent the average and standard deviation for six experiments.

# ^177^Lu extraction efficiency

The ^177^Lu are produced as a result of the internal conversion of ^177m^Lu ions to ^177^Lu ions. The ^177^lu production is defined by equation S1.

| $A_{g}^{t}=A_{m}^{0}\cdot\left( \frac{\lambda_{g}}{{\lambda_{g}-\lambda}_{m}} \right)\cdot\left[ {exp}^{-\lambda_{m.}.t}- {exp}^{-\lambda_{g}.t} \right]\cdot B.R\cdot P.I.C.$ | Equation S1 |
| --- | --- |

where $A_{m}^{0}$ = Initial activity of ^177m^Lu before elution, $\lambda_{g}, \lambda_{m}$ = decay constants of ^177^Lu, ^177m^Lu respectively,$A_{g}^{t}$ = activity of ^177^Lu at time t, B.R = branching ratio for ^177m^Lu to ^177^Lu decay, 21.4% ^26^, P.I.C = probability of internal conversion, 96.8% ^7^,

The growth in the amount of ^177^Lu ions with the increase in the ^177^Lu accumulation period is shown in Figure S2.

|  |
| --- |

Figure S2: The amount of ^177^Lu produced from 1 MBq of ^177m^Lu for different ^177^Lu accumulation period as calculated by using equation 1.

The efficiency of ^177^Lu collection after ^177m^Lu/ ^177^Lu separation is defined as the ratio of the collected ^177^Lu activity in the organic phase divided by the theoretically produced ^177^Lu activity during the accumulation time. It is represented by the equation S2 below;

| $efficiency\left( \% \right)= \frac{A_{g}^{t} \left( collected \right)*({\frac{V_{total}}{V_{collected}}})}{A_{g}^{t}}\cdot100$ | Equation S2 |
| --- | --- |

where $A_{g}^{t}$is the total amount of ^177^Lu produced after an accumulation time t (as defined in equation 1), $A_{g}^{t} \left( collected \right)$ is the ^177^Lu activity measured in the organic phase after LLE, V_total_ and V_collected_ are the total organic volumes and the organic fraction collected after SE respectively, t = time of ^177^Lu separation (7 days).

# ^177^Lu/ ^177m^ Lu activity ratios with time after Liquid- Liquid Extraction

|  |
| --- |

Figure S3: The ^177^Lu/ ^177m^Lu activity ratio obtained at different elution time when the LLE is performed with [^177m^Lu]Lu-DOTA complex synthesized in a molar ratio 1:4. The data points represent the experimentally observed ratios, while the dotted line represents the expected activity ratios with 60% ^177^Lu extraction efficiency and 0.002% ^177m^Lu ions leakage.
